# Pamidronate‐Induced Clinical Remission in Chronic Non‐bacterial Osteomyelitis Is Associated with Reduced Vγ9Vδ2 T‐Cell Receptor Expression

**DOI:** 10.1002/eji.202451609

**Published:** 2025-04-21

**Authors:** Lily Watson, Athimalaipet V Ramanan, Elizabeth Oliver, Francisca Segers, Gareth W. Jones, Christine Chew, Anu Goenka

**Affiliations:** ^1^ Bristol Medical School University of Bristol Bristol UK; ^2^ Paediatric Rheumatology Bristol Royal Hospital for Children Bristol UK; ^3^ Translational Health Sciences University of Bristol Bristol UK; ^4^ School of Biological Sciences University of Bristol Bristol UK; ^5^ Paediatric Immunology and Infectious Diseases Bristol Royal Hospital for Children Bristol UK

**Keywords:** chronic non‐bacterial osteomyelitis, gamma‐delta T cells, pamidronate, RNA sequencing

## Abstract

In children with chronic non‐bacterial osteomyelitis, clinical and transcriptional changes in peripheral blood were examined after pamidronate treatment. Clinically effective treatment with pamidronate was associated with reduced expression of two genes (*TRDV2* and *TRGV9*) that encode the subunits of the Vγ9Vδ2 T‐cell receptor.

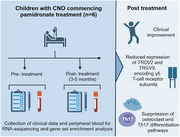

**Abbreviations**
CNO: chronic non‐bacterial osteomyelitis
TCR: T‐cell receptor
γδ: gamma‐delta

Chronic non‐bacterial osteomyelitis (CNO) is an under‐recognised disorder of sterile bone inflammation associated with significant morbidity in affected children and adolescents. Disease onset typically occurs between 7 and 12 years of age and is characterised by bone pain and swelling that can significantly impact quality of life [1]. The annual incidence of CNO is estimated as 6.5 cases per 1,000,000, but this is likely an underestimate and the true incidence may be comparable to bacterial osteomyelitis [2]. Clinician under‐recognition and an absence of disease‐specific biomarkers for CNO contribute to significant diagnostic delay. The aetiology of CNO is unknown which has hampered the development of targeted treatments and diagnostics. Improving our understanding of CNO pathogenesis was recently identified as a research priority by patients, their families and clinicians [3]. Dysregulated immune pathways have been inconsistently identified in studies of similar monogenic disorders (e.g., Majeed syndrome), mouse models and patients with CNO [4].

Treatment with pamidronate (a bisphosphonate drug) is highly effective in many patients, with complete remission in 54% of children after 3 months and the absence of symptom flares in 80% after 12 months [5]. Despite its beneficial impact on disease trajectory, the mechanism of action of pamidronate in CNO is unclear. The antiresorptive effects of bisphosphonates on osteoclasts are well‐recognised, but immunomodulatory roles are emerging including inhibition of macrophage migration, cytokine secretion and Th17 polarisation [5, 6]. To better understand pamidronate's effect in CNO and elicit potential pathogenic pathways, we performed a before‐after whole blood transcriptome analysis of children with CNO treated with pamidronate.

Six children (five female, one male) with median (range) age of 8 (5–12 years) were identified with recently‐diagnosed CNO requiring pamidronate treatment Table 1, (Supporting Information). There was no relevant past medical history except celiac disease in one child. At initial presentation, painful sites included: jaw (2/6 children), hip (2/6), knee (1/6), clavicle (1/6), thigh (1/6), and back (1/6). All children had received an alternate initial diagnosis, and the median (range) duration between initial presentation and CNO diagnosis was 12 (3–41) months. *HLA‐B27* was positive in one (17%) child. Imaging demonstrated multifocal disease in five (83%) children. No children had previously received treatment with corticosteroids, disease‐modifying anti‐rheumatic drugs, biologics or pamidronate. Clinical metrics of disease activity (visual analogue scales of pain at rest/exercise) were collected immediately before the first cycle of pamidronate treatment and 3–5 months after [1]. The cohort exhibited an improvement in disease activity measures post‐pamidronate, except for one patient who was clinically asymptomatic pre‐treatment and remained so post‐treatment (Figure 1A).

**TABLE 1:** Baseline demographic data.

Case	Sex	Age at blood sample immediately prior to pamidronate (years)	Ethnicity	Past medical history	Family History	Age at symptom onset (years)	Presenting site(s)	Initial diagnosis	Duration of symptom onset to diagnosis (months)	Pre‐pamidronate treatment	Bone biopsy result	*HLA ‐B27*	MRI findings at diagnosis	Indication for pamidronate
A	F	12	White British	Nil	Nil	10	Mandible	Garre's osteomyelitis	31	Dental extraction, amoxicillin‐clavulanic acid, paracetamol, ibuprofen	Proliferative periostitis	Neg	Multifocal	Pain + cosmetic
B	F	8	White British	Nil	Brother: alopecia areata	7	Knee	Erdheim–Chester disease	12	Naproxen	ND	Neg	Multifocal	Pain + multifocal
C	F	15	White Mixed	Nil	Mother: psoriasis​	12	Mandible	Infective osteomyelitis	41	Doxycycline	Reactive fibrous lesion	Pos	Unifocal	Pain + cosmetic
D	M	12	White British	Nil	Nil	12	Clavicle	Traumatic fracture	3	Nil	ND	Neg	Multifocal	Pain + multifocal
E	F	8	White British	Nil	Maternal grandfather: psoriasis; maternal grandmother: rheumatoid arthritis	7	Femur, hip, lower back	Osteoid osteoma	13	Naproxen, paracetamol	ND	Neg	Multifocal	Pain + spinal
F	F	6	White British	Celiac disease	Maternal aunt: congenital hypothyroidism	5	Hip	Infective osteomyelitis	6	Naproxen, paracetamol	Nil abnormal	Neg	Multifocal	Spinal

Abbreviations: F, female; M, male; MRI, magnetic resonance imaging; ND, not done

**FIGURE 1:**
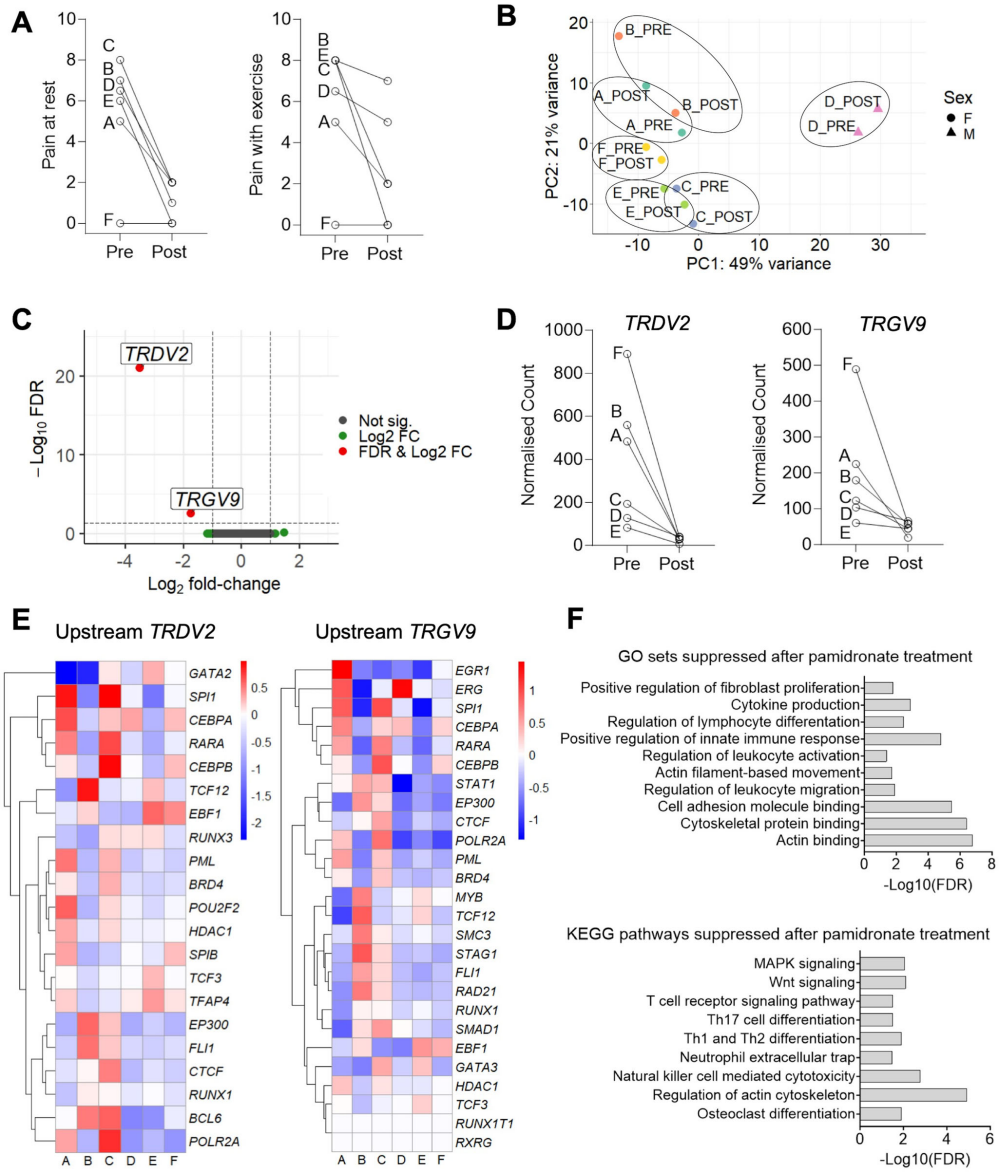
Clinical metrics of disease activity and transcriptional differences pre‐ and post‐pamidronate. (A) Pain at rest visual analogue‐scale (VAS), and pain with exercise VAS, scored 0–10 with 10 being worst pain. (B) Principal component analysis with ellipse demarcating pre‐ and post‐pamidronate samples from the same patient. (C) Volcano plot of differentially expressed genes in post‐pamidronate samples compared with pre‐pamidronate counterparts, with red points representing genes meeting a threshold of log2 fold‐change> 2 and FDR < 0.05. (D) Normalised counts per child for differentially expressed genes. (E) Heatmaps showing gene expression log2 fold‐change of upstream regulators of *TRDV2* and *TRGV9* in post‐treatment samples compared with pre‐treatment samples. (F) KEGG and GO terms identified by gene set enrichment analysis comparing expression pre‐ and post‐treatment selected by biological relevance.

The transcriptional profile of peripheral blood before and after the first cycle of pamidronate treatment was compared with RNA‐seq. Principal Component Analysis revealed that pre‐ and post‐pamidronate gene expression clustered together within the same individual, and the majority of variance was observed between different individuals (Figure 1B). Differential gene expression analysis identified significantly reduced expression of *TRDV2* (log2 fold‐change −3.5, false discovery rate FDR < 0.001) and *TRGV9* (log2 fold‐change −1.8, FDR < 0.003) in post‐pamidronate samples relative to their pre‐pamidronate counterparts (Figure 1C,D). Upstream regulators of differentially regulated genes in blood were predicted using hTFtarget. There was relatively reduced expression of upstream regulators of *TRDV2* and *TRGV9* in post‐treatment samples, relative to their pre‐treatment counterparts (Figure 1E). Gene set enrichment analysis not restricted to differentially expressed genes identified suppression of multiple pathways (Figure 1F), including Th17 cell differentiation (normalised enrichment score NES = −1.65, FDR < 0.05) and osteoclast differentiation (NES = −1.64, FDR < 0.05).

Here, we describe the first gene expression data of children with CNO before and after clinically effective treatment with pamidronate. Strikingly, we identify significantly reduced expression of just two genes (*TRDV2* and *TRGV9*) that encode subunits of the γδ T‐cell receptor (TCR) expressed by γδ T cells. γδ T cells are enriched in several tissues, including skin, intestine, and lung. While the function of γδ T cells is less well‐characterised than αβ T cells, they are thought to play important roles in tissue homeostasis and immune surveillance [7]. While eight δ and seven γ variable genes exist, *TRDV2* and *TRGV9* are selectively expressed by γ9δ2 T cells, the predominant γδ subset in peripheral blood [7].

Our data merits further investigation of the role of γδ T cells, given that *TRDV2* and *TRGV9*: (1) were the only two genes that were significantly and highly differentially expressed; (2) are independently regulated, and upstream genes exhibited reduced expression following pamidronate treatment; and (3) biological plausibility. Pamidronate is known to induce phosphoantigen signalling through the γδ TCR [8]. γδ T cells and pamidronate also both regulate osteoclast function, which includes bone resorption that may play a role in CNO osteolytic lesions. Phosphoantigen‐stimulated γδ T cells inhibit osteoclastogenesis and bone resorption in vitro [9] and our data indicates reduced expression of genes involved in the osteoclast differentiation pathway following pamidronate treatment.

We also found that transcriptional changes post‐pamidronate were associated with a reduction in genes involved in the Th17 cell differentiation pathway. Th17 cells have been linked to CNO pathogenesis given elevated serum IL‐6 concentrations (a key Th17‐polarising cytokine) in CNO and clinical overlap between CNO and other IL‐17‐mediated disorders, including psoriasis, palmoplantar pustulosis, and ankylosing spondylitis [10]. Of note, osteoclastogenesis is regulated by Th17 cytokines [10].

This study was limited by transcriptional profiling at a single time‐point post‐pamidronate. While sufficient to observe a clinically meaningful effect of pamidronate, this strategy could have missed important changes in gene expression before and after this time point. Another limitation of our data is that it describes gene changes in peripheral blood, rather than the affected bony site(s). Despite this, we find potentially biologically and clinically relevant changes in gene expression in peripheral blood, suggesting peripheral blood may yet yield further information on CNO pathogenesis and biomarkers. We hypothesise that pamidronate acts by the modulation of γδ TCR expression and regulation of osteoclast activity, as indicated by the suppression of gene expression in pathways related to osteoclastogenesis and Th17 differentiation. While our RNA‐seq findings indicate suppressed γδ TCR expression and Th17 differentiation, validation of these effects at the protein expression and functional levels is necessary. Future studies should also aim to confirm our findings in a larger cohort, including assessment of IL‐17 production and Th17‐associated markers to confirm the suppression of Th17 polarisation by pamidronate. (Supporting Information)

## Author Contributions

Lily Watson, Christine Chew and Anu Goenka wrote the original draft of the manuscript, and all authors contributed to and approved the article for publication. Christine Chew, Athimalaipet V Ramanan and Anu Goenka conceptualised and designed the study. Christine Chew, Lily Watson and Elizabeth Oliver performed the laboratory work. Francisca Segers analysed the study. Francisca Segers and Lily Watson visualised the study. Christine Chew and Anu Goenka supervised the study.

## Conflicts of Interest

AVR discloses speaker fees/honoraria/consulting fees from the following: Abbvie, Eli Lilly, Pfizer, Novartis, Roche, SOBI, and UCB. The remaining authors declare no conflicts of interest.

### Peer Review

The peer review history for this article is available at https://publons.com/publon/10.1002/eji.202451609.

## Supporting information



Supporting Information

## Data Availability

The RNA sequencing data that support the findings of this study are openly available from the NCBI Sequence Read Archive (SRA) database at https://www.ncbi.nlm.nih.gov/sra/PRJNA1128176, BioProject accession number: PRJNA1128176.
