# Supplementary material for: Pamidronate‐Induced Clinical Remission in Chronic Non‐bacterial Osteomyelitis Is Associated with Reduced Vγ9Vδ2 T‐Cell Receptor Expression
Source: Eur J Immunol. 2025 Apr 21;55(4):e202451609. doi: 10.1002/eji.202451609 (PMC12010949; doi:10.1002/eji.202451609)
Supplement: Supplementary file 1 — Supporting Information [file EJI-55-e202451609-s001.docx]

**Pamidronate-induced clinical remission in chronic non-bacterial osteomyelitis is associated with reduced Vγ9Vδ2 T-cell receptor expression**

**Supplemental Data**

Lily Watson BMBCh^a,b^, Athimalaipet V Ramanan FMedSci^b,c^, Elizabeth Oliver BSc^a^, Francisca Segers PhD^d^, Gareth W. Jones PhD^a^, Christine Chew PhD^a,b^ *, Anu Goenka PhD^a,e^ *

*contributed equally

^a^ Bristol Medical School, University of Bristol, Bristol, UK.
^b^ Paediatric Rheumatology, Bristol Royal Hospital for Children, Bristol, UK.
^c^ Translational Health Sciences, University of Bristol, Bristol, UK.
^d^ School of Biological Sciences, University of Bristol, Bristol, UK.
^e^ Paediatric Immunology and Infectious Diseases, Bristol Royal Hospital for Children, Bristol, UK.

Correspondence to Dr Anu Goenka [anu.goenka@bristol.ac.uk](mailto:anu.goenka@bristol.ac.uk)

**Material and Methods**

*Cohort and sampling*

Children aged 16 years or younger with a diagnosis of CNO who met the Bristol Criteria [1] and were due to start intravenous pamidronate treatment at the Bristol Royal Hospital for Children between August 2022 and November 2023 were included. Informed consent from parents and samples were obtained under approval of the Bristol Biobank (NHS REC 20/WA/0273). Peripheral blood (2.5mL) was collected in PAXgenes bottles (PreAnalytiX/Qiagen) immediately before and 3-5 months after first pamidronate cycle. Samples were stored at room temperature for up to 6h and then frozen at -20°C (<48 hours) before transfer to -70°C. Clinical metrics of disease activity (visual analogue-scales of pain at rest/exercise) were recorded [2].

*RNA sequencing*

Total RNA was extracted and purified from blood samples using the PAXgene Blood RNA Kit (PreAnalytiX/Qiagen) as per manufacturer’s instructions. RNA quality was assessed by RNA TapeStation (Agilent) and Nanodrop (Thermo Scientific). Libraries were prepared with the Illumina Stranded mRNA library preparation kit (Illumina). Paired-end sequences from NextSeq500 sequencer (Illumina) were quality trimmed and adaptor sequencers were removed with Fastp V0.12.4 [3].

*Analysis*

Reads were mapped to the human reference genome build GRCh38 with STAR V2.7.9a [4] and counts per gene were obtained by HTSeq-count V0.11.3 [5]. Normalisation, principal component analysis, and differential expression was calculated with with DESeq2 V1.40.2 [6]. Participant identity was included in statistical modelling to account for repeated sampling of the same individual. Adjusted *p*-values were corrected for multiple testing by False Discovery Rate (FDR) method. Gene set enrichment analysis not restricted to differentially expressed genes was performed using Kyoto Encyclopedia of Genes and Genomes (KEGG) and Gene Ontology (GO) terms in clusterProfiler V4.8.3 [7] with org.Hs.eg.db V3.17.0 [8]. Upstream regulators of differentially regulated genes in blood were predicted using hTFtarget [9]. Fold-change pre- vs post-pamidronate for upstream genes was calculated. Figures were created in GraphPad Prism 10 and R Studio. RNA sequencing data are available from the NCBI Sequence Read Archive (SRA) database with the following BioProject accession number: PRJNA1128176.

**Ethics Approval Statement**

Informed consent from parents was obtained under approval of the Bristol Biobank (NHS REC 20/WA/0273).

**Supplemental References**

[1] M. R. Roderick, R. Shah, V. Rogers, A. Finn, A. Ramanan. Chronic recurrent multifocal osteomyelitis (CRMO) - advancing the diagnosis. Pediatr Rheumatol Online J. 14 (2016) 47. https://doi.org/10.1186/s12969-016-0109-1.

[2] N. Julia, R. Katharina, G. Hermann, H. W. Annette, M. Henner, H. Helge, H. Christine. Physical activity and health-related quality of life in chronic non-bacterial osteomyelitis. Pediatr Rheumatol Online J. 17 (2019) 45. https://doi.org/10.1186/s12969-019-0351-4.

[3] S. Chen, Y. Zhou, Y. Chen, J. Gu. fastp: an ultra-fast all-in-one FASTQ preprocessor. Bioinformatics. 34 (2018) i884-i90. https://doi.org/10.1093/bioinformatics/bty560.

[4] A. Dobin, C. A. Davis, F. Schlesinger, J. Drenkow, C. Zaleski, S. Jha*,* P. Batut, M. Chaisson, T. R. Gingeras*.* STAR: ultrafast universal RNA-seq aligner. Bioinformatics. 29 (2013) 15-21. https://doi.org/10.1093/bioinformatics/bts635.

[5] S. Anders, P. T. Pyl, W. Huber. HTSeq--a Python framework to work with high-throughput sequencing data. Bioinformatics. 31 (2015) 166-9. https://doi.org/ 10.1093/bioinformatics/btu638.

[6] M. I. Love, W. Huber, S. Anders. Moderated estimation of fold change and dispersion for RNA-seq data with DESeq2. Genome Biol. 15 (2014) 550. https://doi.org/10.1186/s13059-014-0550-8.

[7] T. Wu, E. Hu, S. Xu, M. Chen, P. Guo, Z. Dai*,* T. Feng, L. Zhou, W. Tang, L. Zhan, X. Fu, S. Liu, X. Bo, G. Yu*.* clusterProfiler 4.0: A universal enrichment tool for interpreting omics data. Innovation (Camb). 2 (2021) 100141. https://doi.org/10.1016/j.xinn.2021.100141.

[8] C. M. org.Hs.eg.db: Genome wide annotation for Human. R package R package R package 2023, p. R package

[9] Q. Zhang, W. Liu, H. M. Zhang, G. Y. Xie, Y. R. Miao, M. Xia, A. Y. Guo. hTFtarget: A Comprehensive Database for Regulations of Human Transcription Factors and Their Targets. Genomics Proteomics Bioinformatics. 18 (2020) 120-8. https://doi.org/10.1016/j.gpb.2019.09.006.
